# Urgent Transcatheter Mitral Edge‐to‐Edge Repair Is Associated With Worse in‐Hospital Outcomes: A Nationwide Analysis

**DOI:** 10.1002/clc.70067

**Published:** 2025-03-10

**Authors:** Carlos Diaz‐Arocutipa, Cesar Joel Benites‐Moya, Javier Torres‐Valencia, Adhya Mehta, Lourdes Vicent

**Affiliations:** ^1^ Unidad de Revisiones Sistemáticas y Meta‐análisis (URSIGET), Vicerrectorado de Investigación Universidad San Ignacio de Loyola Lima Peru; ^2^ Jacobi Medical Center, Albert Einstein College of Medicine Bronx New York USA; ^3^ Department of Cardiology Hospital Nacional Edgardo Rebagliati Martins Lima Peru; ^4^ Cardiology Department Hospital Universitario 12 de Octubre Madrid Spain

**Keywords:** MitraClip, transcatheter mitral edge‐to‐edge repair, urgent

## Abstract

**Background:**

To assess in‐hospital outcomes in patients undergoing urgent versus non‐urgent transcatheter mitral edge‐to‐edge repair (TEER).

**Methods:**

We used the NIS database 2016−2019 to include admissions who underwent TEER. Inverse probability of treatment weighting (IPTW) was used to compare urgent versus non‐urgent groups.

**Results:**

A total of 29 730 weighted admissions were included, of whom 21.6% were urgent admissions. Urgent admissions had a higher risk of in‐hospital mortality (risk ratio [RR] 3.67, 95% confidence interval [CI] 2.39−5.62), cardiogenic shock (RR 4.95, 95% CI 3.73−6.57), intra‐aortic balloon pump (RR 3.97, 95% CI 2.53−6.23), percutaneous ventricular assist device (RR 17.24, 95% CI 6.37−46.66), mechanical ventilation (RR 3.79, 95% CI 2.80−5.11), acute stroke (RR 2.56, 95% CI 1.32−4.97), in‐hospital cardiac arrest (RR 2.25, 95% CI 1.08−4.69), major bleeding (RR 5.18, 95% CI 2.97−9.06), increased length of stay (6 vs. 2 days, *p* < 0.001), and higher total costs ($229 160 vs. $164 653, *p* < 0.01) compared to non‐urgent admissions. There was no difference between both groups for renal replacement therapy and pericardial complication.

**Conclusion:**

Our results suggest that urgent TEER implantation was associated with an increased risk of in‐hospital death and other short‐term complications.

## Graphical abstract

This study aimed to assess in‐hospital outcomes in patients undergoing urgent versus non‐urgent transcatheter mitral edge‐to‐edge repair, using the National Inpatient Sample database 2016−2019. A total of 29 730 weighted admissions were included. Urgent admissions had a higher risk of in‐hospital mortality, cardiogenic shock, intra‐aortic balloon pump, percutaneous ventricular assist device, mechanical ventilation, acute stroke, in‐hospital cardiac arrest, major bleeding, increased length of stay, and higher total costs compared to non‐urgent admissions.

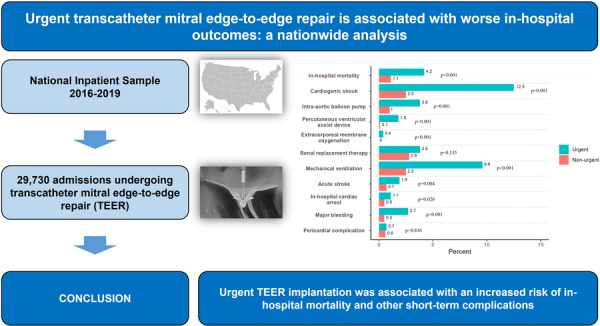

## 1 Introduction

Mitral regurgitation (MR) is one of the most common valvular heart diseases in the general population [1], particularly in patients with heart failure [2]. MR is a factor associated with poor prognosis, with increased mortality and hospital readmissions, especially in the presence of reduced left ventricular ejection fraction [3].

Transcatheter mitral valve edge‐to‐edge repair (TEER) has emerged as a promising alternative to surgery for patients with severe primary or secondary MR and favorable valve anatomy, particularly those considered high risk or ineligible for conventional surgery due to advanced age, significant comorbidities or anatomical constraints [4, 5]. This lesser invasive approach involves the percutaneous placement of a mitral valve clip to reduce regurgitation by approximating the mitral valve leaflets [6].

While the majority of TEER procedures are planned and performed electively to optimize patient selection and preparation [7, 8], there are scenarios where urgent intervention is required, such as acute decompensation leading to severe MR‐related symptoms, acute exacerbation of heart failure or hemodynamic instability [9]. Despite the increasing use of TEER, there is a paucity of studies in the literature specifically addressing the outcomes of urgent TEER procedures.

Previous research has highlighted the poorer outcomes associated with urgent invasive cardiovascular procedures compared to elective procedures [10]. However, the unique considerations and outcomes associated with urgent TEER remain less explored.

Using a robust data set that includes a wide range of patient demographics, clinical characteristics and procedural details, we aim to elucidate the impact of urgency on TEER outcomes. Therefore, our study aimed to compare in‐hospital outcomes in patients undergoing urgent versus non‐urgent TEER using a contemporary nationwide database.

## 2 Methods

We conducted a retrospective study using the National Inpatient Sample (NIS) database during the period 2016−2019. The NIS is a publicly available database of the Health Care Utilization Project, which contains data for 20% of discharge records from community hospitals across the United States. Admissions of adults who underwent in‐hospital TEER using the appropriate ICD‐10 procedure codes were included (Supporting Information S1: Table 1). Admissions with missing data for covariates were excluded. Patients were divided into two groups, urgent and non‐urgent TEER, for comparison. For the definition of the type of TEER procedure (urgent vs. non‐urgent), we used the variable “ELECTIVE” from the NIS database which has two categories: non‐elective versus elective admission.

The primary outcome was in‐hospital mortality, and the secondary outcomes were cardiogenic shock, pulmonary artery catheterization, intra‐aortic balloon pump (IABP), percutaneous ventricular assist device (PVAD), extracorporeal membrane oxygenation (ECMO), renal replacement therapy, mechanical ventilation, acute stroke, major bleeding, pericardial complication, length of hospital stay, and total charges (Supporting Information S1: Table 1). Sociodemographic characteristics, comorbidities (based on Elixhauser Comorbidity Index), and hospital characteristics were reported.

**Figure 1:**
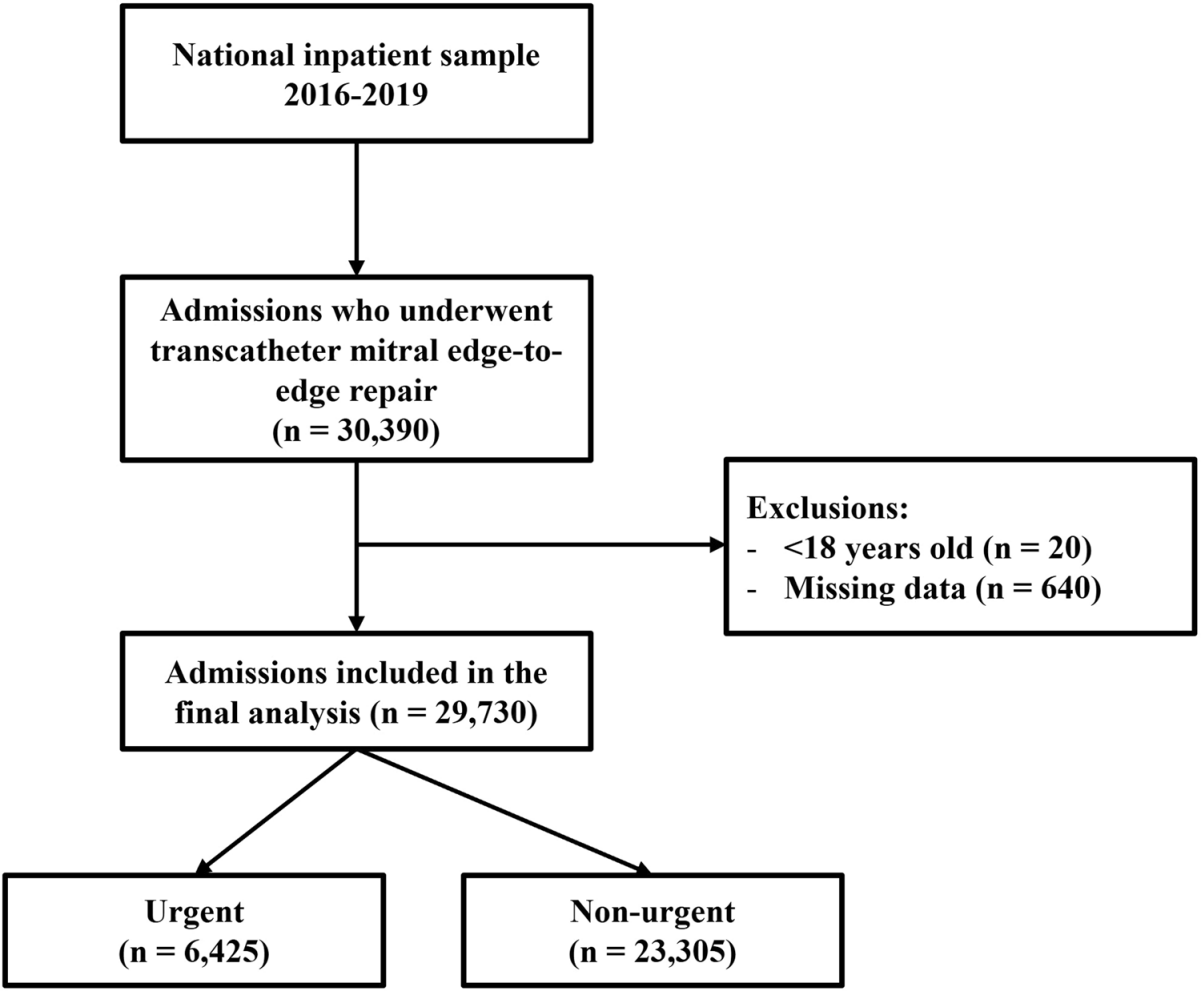
Flow diagram for the selection of study participants.

Categorical variables were expressed as frequencies and percentages and continuous variables as median (interquartile range [IQR]). A chi‐squared test with Rao & Scott's second‐order correction and Wilcoxon rank‐sum test were used to compare categorical and continuous variables, respectively. Inverse probability of treatment weighting (IPTW) was used to assess the differences between urgent and non‐urgent groups, balancing demographics, comorbidities, and hospital characteristics. The balance of baseline covariates was compared using the standardized mean difference (cut‐off < 0.1 for appropriate balance) (Supporting Information S1: Figure 1). A log‐binomial model was used to estimate the adjusted risk ratios (aRR) with their 95% confidence intervals (CI). In addition, we performed a trend analysis on cases of TEER and urgent admissions, examining quarterly data each year using the Cochran‐Armitage Trend Test. The R 4.3.2 software was used for all analyses, considering a two‐tailed *p* < 0.05 as statistically significant.

## 3 Results

### 3.1 Demographics and Characteristics

This study involved 30 390 weighted admissions of adults who underwent TEER. Of these, 29 730 were included in the final analysis, with 6425 (21.6%) classified as urgent admissions (Figure 1). The median age of the cohort was 79 years (IQR 71−85), with 45.8% being female and 77.7% identifying as white (Table 1). The most prevalent comorbidities among this cohort were hypertension (81.8%), atrial fibrillation (59.4%), and dyslipidemia (58.6%). Patients with urgent admissions exhibited a higher comorbidity burden, particularly in extracardiac conditions such as renal failure (50.4% vs. 34.2%), diabetes (32.1% vs. 24%), and chronic pulmonary disease (31.1% vs. 22.3%), all of which were statistically significant differences (*p* < 0.001). The median Elixhauser Comorbidity Index was also higher in the urgent admission group (7.00 vs. 5.00, *p* < 0.001).

**Table 1:** Characteristics of included admissions.

Characteristic	Overall	Type of admission		
		Non‐urgent	Urgent	*p* value
Weighted admissions	29 730	23 305	6425	
Age (years)*	79 (71−85)	79 (71−85)	78 (69−85)	0.196
Female sex	13 605.0 (45.8%)	10 580.0 (45.4%)	3025.0 (47.1%)	0.284
Race				< 0.001
White	23 115.0 (77.7%)	18 430.0 (79.1%)	4685.0 (72.9%)	
Black	2375.0 (8.0%)	1740.0 (7.5%)	635.0 (9.9%)	
Hispanic	1695.0 (5.7%)	1185.0 (5.1%)	510.0 (7.9%)	
Other	2545.0 (8.6%)	1950.0 (8.4%)	595.0 (9.3%)	
Household income				0.008
Quartile 1	6420.0 (21.6%)	4830.0 (20.7%)	1590.0 (24.7%)	
Quartile 2	6860.0 (23.1%)	5350.0 (23.0%)	1510.0 (23.5%)	
Quartile 3	8305.0 (27.9%)	6595.0 (28.3%)	1710.0 (26.6%)	
Quartile 4	8145.0 (27.4%)	6530.0 (28.0%)	1615.0 (25.1%)	
Hypertension	24 320.0 (81.8%)	18 850.0 (80.9%)	5470.0 (85.1%)	< 0.001
Atrial fibrillation	17 650.0 (59.4%)	13 680.0 (58.7%)	3970.0 (61.8%)	0.046
Dyslipidemia	17 420.0 (58.6%)	13,870.0 (59.5%)	3550.0 (55.3%)	0.006
Renal failure	11 200.0 (37.7%)	7965.0 (34.2%)	3235.0 (50.4%)	< 0.001
Diabetes	7655.0 (25.7%)	5590.0 (24.0%)	2065.0 (32.1%)	< 0.001
Chronic pulmonary disease	7640.0 (25.7%)	5640.0 (24.2%)	2000.0 (31.1%)	< 0.001
Peripheral vascular disorders	6720.0 (22.6%)	5205.0 (22.3%)	1515.0 (23.6%)	0.345
Previous PCI	5600.0 (18.8%)	4520.0 (19.4%)	1080.0 (16.8%)	0.036
Previous CABG	5585.0 (18.8%)	4555.0 (19.5%)	1030.0 (16.0%)	0.004
Previous myocardial infarction	4370.0 (14.7%)	3400.0 (14.6%)	970.0 (15.1%)	0.649
Previous stroke	3500.0 (11.8%)	2715.0 (11.6%)	785.0 (12.2%)	0.576
Previous ICD	3370.0 (11.3%)	2680.0 (11.5%)	690.0 (10.7%)	0.447
Previous pacemaker	3165.0 (10.6%)	2515.0 (10.8%)	650.0 (10.1%)	0.487
Elixhauser Comorbidity Index^a^	6.00 (4.00−7.00)	5.00 (4.00−7.00)	7.00 (5.00−8.00)	< 0.001
Admission on weekend	1,135.0 (3.8%)	170.0 (0.7%)	965.0 (15.0%)	< 0.001
Expected primary payer				< 0.001
Medicare	24 585.0 (82.7%)	19 285.0 (82.8%)	5300.0 (82.5%)	
Medicaid	750.0 (2.5%)	450.0 (1.9%)	300.0 (4.7%)	
Private	3775.0 (12.7%)	3155.0 (13.5%)	620.0 (9.6%)	
Other	620.0 (2.1%)	415.0 (1.8%)	205.0 (3.2%)	
Bed size of hospital				< 0.001
Small	1990.0 (6.7%)	1440.0 (6.2%)	550.0 (8.6%)	
Medium	5630.0 (18.9%)	4615.0 (19.8%)	1015.0 (15.8%)	
Large	22 110.0 (74.4%)	17 250.0 (74.0%)	4860.0 (75.6%)	
Location of hospital				0.341
Rural	160.0 (0.5%)	140.0 (0.6%)	20.0 (0.3%)	
Urban non‐teaching	2200.0 (7.4%)	1755.0 (7.5%)	445.0 (6.9%)	
Urban teaching	27 370.0 (92.1%)	21 410.0 (91.9%)	5960.0 (92.8%)	
Region of hospital				< 0.001
Northeast	5430.0 (18.3%)	4050.0 (17.4%)	1380.0 (21.5%)	
Midwest	5920.0 (19.9%)	5080.0 (21.8%)	840.0 (13.1%)	
South	10 630.0 (35.8%)	8270.0 (35.5%)	2,360.0 (36.7%)	
West	7750.0 (26.1%)	5905.0 (25.3%)	1,845.0 (28.7%)	
Ownership of hospital				< 0.001
Government, nonfederal	2670.0 (9.0%)	2110.0 (9.1%)	560.0 (8.7%)	
Private, non‐profit	24 045.0 (80.9%)	19 205.0 (82.4%)	4840.0 (75.3%)	
Private, investor‐own	3015.0 (10.1%)	1990.0 (8.5%)	1025.0 (16.0%)	
Transfer out indicator				< 0.001
Not a transfer	26 790.0 (90.1%)	21 825.0 (93.6%)	4965.0 (77.3%)	
Different acute care hospital	160.0 (0.5%)	60.0 (0.3%)	100.0 (1.6%)	
Another type of health facility	2780.0 (9.4%)	1420.0 (6.1%)	1360.0 (21.2%)	

Abbreviations: PCI = percutaneous coronary intervention, CABG = coronary artery bypass grafting, ICD = implantable cardioverter‐defibrillator.

a Median (interquartile range).

### 3.2 Procedures and Outcomes

The median length of stay for all admissions was 2 days (IQR 1−4), with a majority of procedures conducted in large urban teaching hospitals. Notably, a higher proportion of urgent admissions were transferred to another hospital (21.2% vs. 6.1%, *p* < 0.001) (Table 2).

**Table 2:** Crude and weighted analysis for all outcomes.

Characteristic	Overall	Crude			Weighted		
		Non‐urgent	Urgent	*p* value	Non‐urgent	Urgent	*p* value
In‐hospital mortality	1.8%	1.0%	4.8%	< 0.001	1.1%	4.2%	< 0.001
Cardiogenic shock	5.1%	2.3%	15.3%	< 0.001	2.5%	12.5%	< 0.001
Pulmonary artery catheterization	12.5%	9.4%	23.8%	< 0.001	9.8%	21.5%	< 0.001
Intra‐aortic balloon pump	1.7%	0.9%	4.7%	< 0.001	1.0%	3.8%	< 0.001
Percutaneous ventricular assist device	0.5%	0.1%	1.9%	< 0.001	0.1%	1.8%	< 0.001
Extracorporeal membrane oxygenation	0.2%	0.0%	0.6%	< 0.001	0.0%	0.4%	< 0.001
Renal replacement therapy	2.7%	2.1%	4.7%	< 0.001	2.8%	3.8%	0.135
Mechanical ventilation	4.3%	2.3%	11.8%	< 0.001	2.5%	9.6%	< 0.001
Acute stroke	0.9%	0.7%	1.6%	0.004	0.7%	1.9%	0.004
In‐hospital cardiac arrest	0.6%	0.5%	1.2%	0.005	0.5%	1.1%	0.026
Major bleeding	1.0%	0.5%	2.8%	< 0.001	0.5%	2.7%	< 0.001
Pericardial complication	0.8%	0.7%	1.1%	0.120	0.6%	0.7%	0.836
Length of hospital stay (days)	2.0 (1.0−4.0)	2.0 (1.0−3.0)	7.0 (3.0−14.0)	< 0.001	2 (1−3)	6 (2−12)	< 0.001
Total charges ($)	176 600 (125 295−268 242)	159 482 (119 688−236 155)	256 961 (171 691−398 663)	< 0.001	164 653 (121 281−243 251)	229 160 (154 865−344 221)	< 0.001

**Figure 2:**
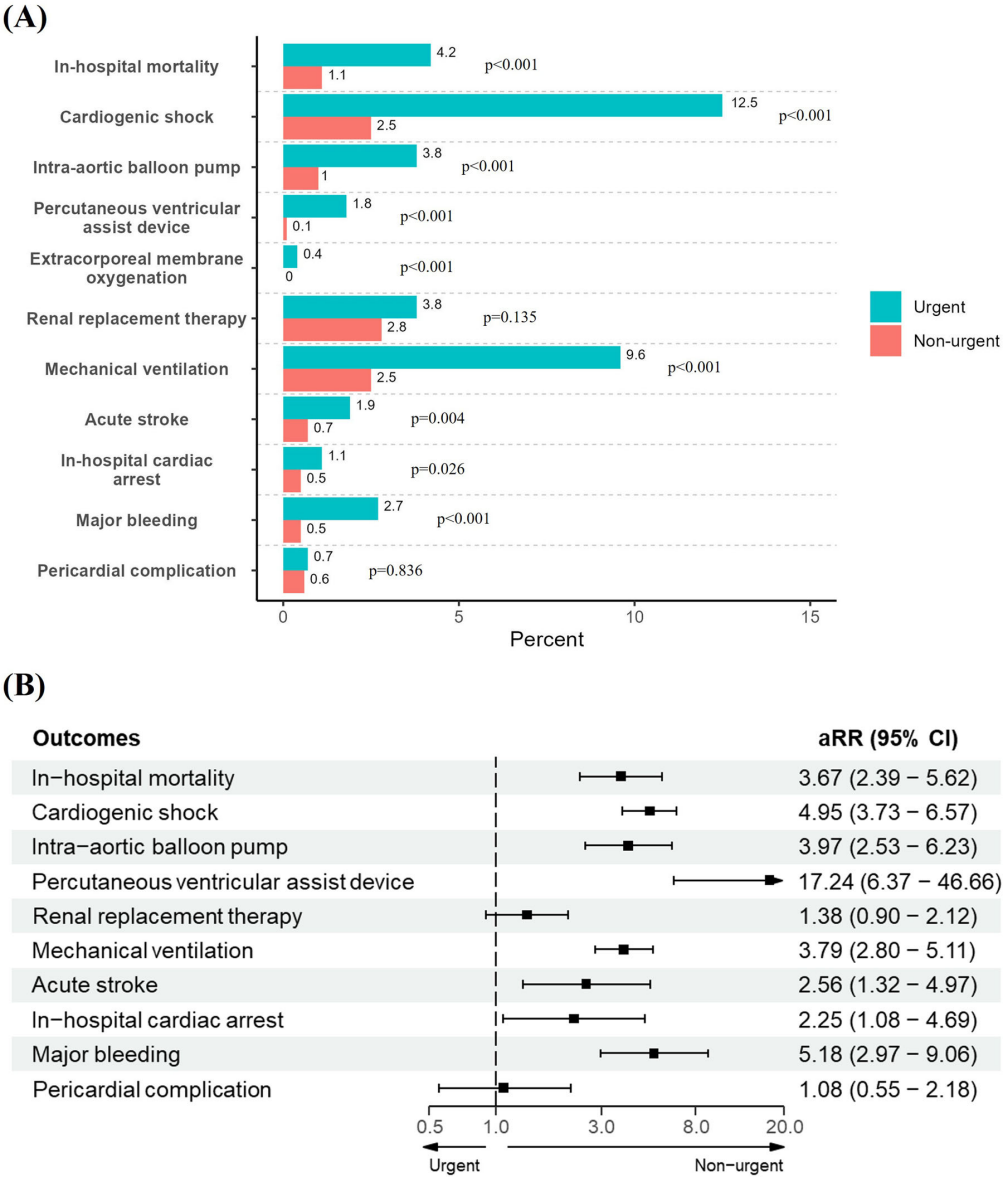
(A) Adjusted proportions and (B) adjusted relative risks for primary and secondary outcomes according to urgent and non‐urgent groups. aRR = adjusted relative risk; CI = confidence interval.

The overall in‐hospital mortality rate for TEER admissions was 1.8%, with urgent admissions demonstrating a significantly higher in‐hospital mortality rate after IPTW adjustment (aRR 3.67, 95% CI 2.39–5.62) compared to non‐urgent admissions (Table 2, Table 3, and Figure 2). Similarly, patients admitted urgently were at a higher risk of developing cardiogenic shock (aRR 4.95, 95% CI 3.73−6.57), acute stroke (aRR 2.56, 95% CI 1.32−4.97), and compared to non‐urgent admissions, in‐hospital cardiac arrest (aRR 2.25, 95% CI 1.08−4.69) and major bleeding (aRR 5.18, 95% CI 2.97−9.06) were also more frequent among urgent admissions (Table 3 and Figure 2).

**Table 3:** Association between type of TEER procedure and outcomes.

Outcomes	Crude model			Adjusted model^a^		
	RR	95% CI	*p* value	RR	95% CI	*p* value
*In‐hospital mortality*						
Non‐urgent	Ref.			Ref.		
Urgent	4.89	3.36−7.12	< 0.001	3.67	2.39−5.62	< 0.001
*Cardiogenic shock*						
Non‐urgent	Ref.			Ref.		
Urgent	6.81	5.41−8.55	< 0.001	4.95	3.73−6.57	< 0.001
*Pulmonary artery catheterization*						
Non‐urgent	Ref.			Ref.		
Urgent	2.53	2.22−2.89	< 0.001	2.19	1.87−2.57	< 0.001
*Intra‐aortic balloon pump*						
Non‐urgent	Ref.			Ref.		
Urgent	5.40	3.65−7.98	< 0.001	3.97	2.53−6.23	< 0.001
*Percutaneous ventricular assist device*						
Non‐urgent	Ref.			Ref.		
Urgent	18.14	6.95−47.29	< 0.001	17.24	6.37−46.66	< 0.001
*Extracorporeal membrane oxygenation*						
Non‐urgent	Ref.			Ref.		
Urgent	14.51	3.08−68.27	< 0.001	10.93	2.25−53.19	0.003
*Renal replacement therapy*						
Non‐urgent	Ref.			Ref.		
Urgent	2.21	1.62−3.02	< 0.001	1.38	0.90−2.12	0.136
*Mechanical ventilation*						
Non‐urgent	Ref.			Ref.		
Urgent	5.12	4.03−6.51	< 0.001	3.79	2.80−5.11	< 0.001
*Acute stroke*						
Non‐urgent	Ref.			Ref.		
Urgent	2.20	1.27−3.82	0.005	2.56	1.32−4.97	0.005
*In‐hospital cardiac arrest*						
Non‐urgent	Ref.			Ref.		
Urgent	2.47	1.29−4.75	0.007	2.25	1.08−4.69	0.03
*Major bleeding*						
Non‐urgent	Ref.			Ref.		
Urgent	5.22	3.15−8.67	< 0.001	5.18	2.97−9.06	< 0.001
*Pericardial complication*						
Non‐urgent	Ref.			Ref.		
Urgent	1.64	0.87−3.07	0.124	1.08	0.53−2.18	0.836

Abbreviations: CI = confidence interval, RR = risk ratio.

a Adjusted for age, sex, race/ethnicity, household income, comorbidities, weekend admission, expected insurance payer, bed size of hospital, location of hospital, region of hospital, and ownership of hospital.

Furthermore, the utilization of invasive procedures was more common among urgent‐TEER patients, including IABP (aRR 3.97, 95% CI 2.53−6.23), PVAD (aRR 17.24, 95% CI 6.37−46.66), and mechanical ventilation (aRR 3.79, 95% CI 2.80−5.11) (Figure 2). No significant difference was observed between the two groups with respect to renal replacement therapy and pericardial complications (Table 3 and Figure 2). Urgent admissions were associated with longer median length of stay (median 6 vs. 2 days, *p* < 0.001) and higher total costs (median $229 160 vs. $164 653, *p* < 0.01) compared to non‐urgent admissions (Table 2).

A statistically significant increase in the utilization of TEER was observed over time (*p* < 0.001), while the proportion of urgent admissions remained unchanged across the study period (*p* = 0.652) (Supporting Information S1: Figure 2). Likewise, there was no temporal change in the length of hospital stay (*p* = 0.425) and total charges (*p* = 0.950) (Supporting Information S1: Table 2).

## 4 Discussion

In this nationwide study, we found that urgent admissions represented nearly a quarter of all cases of patients undergoing TEER. Inpatients undergoing urgent TEER had a higher risk of in‐hospital mortality, an increased requirement of mechanical circulatory support, and other in‐hospital complications, along with a higher utilization of hospital resources.

MR plays a significant role in the setting of acute decompensated heart failure, both in terms of its frequency and its impact on prognosis [3, 11]. Given the relevance of MR and the risk profile of patients with acute HF, the approach of performing a less invasive therapeutic option such as TEER is of interest [11, 12]. Our study has documented an increased risk of adverse clinical events associated with the performance of TEER procedures in an emergency setting. Similar results were observed in patients undergoing cardiac surgery in terms of a worse outcome of urgent interventions. In this urgent group, a previous study found that those who received surgical mitral valve repair had a somewhat lower risk of mortality and complications than those who underwent mitral valve replacement surgery [13]. The question remains as to whether the prognosis of these patients can truly be improved with TEER in this emergency setting compared to medical treatment alone [14].

Previous studies have shown mixed results for urgent versus non‐urgent TEER in the short‐ and long‐term [15, 16, 17, 18]. Al‐khadra et al. used administrative data and found no significant differences in in‐hospital mortality (4.4% vs. 2.8%, *p* = 0.051) and cardiac complications between the two groups after propensity score matching [15]. Similarly, a study conducted in Spain on 85 patients with degenerative and functional MR reported no differences in mortality, MR reduction, and improvement in the New York Heart Association class at 30 days between urgent and non‐urgent TEER [16]. Furthermore, mortality was similar between both groups at a 2‐year follow‐up (17.6% vs. 25.1%, *p* = 0.864). In contrast, in a more recent NIS cohort (2016−2017), in‐hospital mortality was found to be significantly higher in urgent versus non‐urgent TEER (4.5% vs. 1.6%, *p* < 0.001) [18]. Overall, these discrepancies can be explained by the use of different ICD‐10 codes to define the TEER, the type of analysis employed (crude vs. confounder‐adjusted), the study period considering the recent approval of the MitraClip device and the experience of the operators performing the procedure.

It is reasonable to assume that patients undergoing urgent TEER present a higher‐risk profile compared to those with non‐urgent indications [7, 8, 19]. This higher‐risk profile likely encompasses multiple factors beyond comorbidities alone. The observed increase in mortality and complication rates among patients undergoing urgent TEER may be attributed to the more severe clinical status at the time of intervention. We found that patients requiring urgent TEER often present with acute decompensation of heart failure and significant hemodynamic instability with cardiogenic shock in 12%, necessitating prompt intervention. These clinical factors, including the severity of MR, left ventricular dysfunction, and associated comorbidities may impact procedural outcomes [20, 21]. Factors such as hemodynamic instability, organ dysfunction, and the need for mechanical circulatory support can contribute to increased peri‐procedural risks and post‐procedural complications [13, 17].

## 5 Clinical Implications

It is essential to optimize the clinical situation of patients before the TEER procedure, if possible, particularly in those with urgent admissions. A recent study observed that in patients with MR following acute myocardial infarction, cardiogenic shock was not a factor associated with a worse outcome. This work highlighted the importance of achieving hemodynamic stability as a primary goal before TEER, if this is feasible [22]. Also, a further study demonstrated that patients with cardiogenic shock and MR who underwent TEER exhibited acceptable survival and procedural success [20]. However, it is highly advisable to exercise caution when interpreting these results, as they are likely to be selected cases.

Our study highlights the importance of robust risk stratification tools to identify patients at higher risk of adverse outcomes when undergoing urgent TEER. Factors such as comorbidities, hemodynamic stability, and severity of MR should be carefully evaluated to guide treatment decisions and improve patient's optimization before and after TEER [19, 21].

The optimal timing of interventions is a crucial consideration. The decision to perform TEER urgently versus electively should be informed by a comprehensive assessment of individual patient characteristics, including the severity of symptoms, hemodynamic status, and overall clinical stability. Balancing the potential benefits of early intervention with the inherent risks associated with urgent procedures is crucial. A multidisciplinary approach involving cardiologists, cardiac surgeons, and other specialists is often required for the management of patients with severe MR [23]. It is crucial that these teams work together to conduct a thorough risk assessment, develop an effective treatment plan, and provide comprehensive care following the procedure [24].

The majority of TEER procedures were conducted in large urban teaching hospitals, indicating a concentration of specialized care in these settings. Interestingly, while there was an overall increase in TEER utilization over time, the proportion of urgent admissions remained stable, suggesting consistent patient selection criteria for urgent interventions.

It should be noted that our study has certain limitations, primarily due to the retrospective design and reliance on administrative data. There is a possibility that unmeasured confounding variables may have influenced our findings. Furthermore, important clinical parameters such as MR etiology, echocardiographic or hemodynamic data, specific TEER implant characteristics, and medication usage during hospitalization were not captured in our analysis. Additionally, the short‐term nature of our study precludes assessment of long‐term outcomes.

## 6 Conclusions

In conclusion, adult inpatients undergoing urgent TEER implantation had an increased risk of in‐hospital death and other short‐term complications. However, prospective multicenter studies evaluating long‐term outcomes are required to guide the care of patients with severe MR requiring urgent intervention.

## Author Contributions

Carlos Diaz‐Arocutipa involved in concept/design. Carlos Diaz‐Arocutipa involved in data acquisition. Carlos Diaz‐Arocutipa, Cesar Joel Benites‐Moya, Javier Torres‐Valencia, Adhya Mehta, and Lourdes Vicent involved in data analysis/interpretation. Carlos Diaz‐Arocutipa and Lourdes Vicent drafted the article. Cesar Joel Benites‐Moya, Javier Torres‐Valencia, and Adhya Mehta critically revised the article. Carlos Diaz‐Arocutipa, CBM, Javier Torres‐Valencia, Adhya Mehta, and Lourdes Vicent approved the article.

## Ethics Statement

Not applicable because only information from published studies was used.

## Conflicts of Interest

The authors declare no conflicts of interest.

## Supporting information

Supporting information.

## Data Availability

Data are available in a public, open access repository. National Inpatient Sample is available online at https://hcup-us.ahrq.gov/databases.jsp.
