# Supplementary material for: Urgent Transcatheter Mitral Edge‐to‐Edge Repair Is Associated With Worse in‐Hospital Outcomes: A Nationwide Analysis
Source: Clin Cardiol. 2025 Mar 10;48(3):e70067. doi: 10.1002/clc.70067 (PMC11892689; doi:10.1002/clc.70067)
Supplement: Supplementary file 1 — Supporting information. [file CLC-48-e70067-s001.docx]

**SUPPLEMENTARY DATA**

**Table S1. Search ICD-10 codes**

| **Diagnoses/Procedures** | **Codes** |
| --- | --- |
| Transcatheter mitral valve edge-to-edge repair | 02UG3JZ, 02UG37E, 02UG37Z, 02UG38E, 02UG38Z, 02UG3JE, 02UG3JH, 02UG3KE, 02UG3KZ, 02UG47E, 02UG47Z, 02UG48E, 02UG48Z, 02UG4JE, 02UG4JZ, 02UG4KE, 02UG4KZ |
| Cardiogenic shock | R570, T8111* |
| Pulmonary artery catheterization | 4A023N6, 4A023N8, 4A0239Z, 4A033B3, 4A033J3, 4A03353, 4A13353, 4A133B3, 4A133J3, 4A1239Z, 02HP32Z, 02HP30Z |
| Intra-aortic balloon pump | 5A02110, 5A02210 |
| Percutaneous ventricular assist device | 5A0211D, 5A0221D, 5A02116, 5A02216, 02HA0QZ, 02HA3RJ, 02HA3RS, 02HA3RZ |
| Extracorporeal membrane oxygenation | 5A1522F, 5A1522G, 5A1522H, 5A15223 |
| Renal replacement therapy | 5A1D70Z, 5A1D80Z, 5A1D90Z |
| Mechanical ventilation | 5A1935Z, 5A1945Z, 5A1955Z |
| Acute stroke | I63* |
| Major bleeding | I60*, I61*, I62*, K920, K921, K922 |
| Pericardial complication | I312, I3139, I314 |

**Table S2. Temporal changes for length of hospital stay and total charges**

| Period | Length of hospital stay (days)* | Total charges ($)* |
| --- | --- | --- |
| 2016-I | 3.0 (2.0 - 7.0) | 189,857 (138,045 - 262,757) |
| 2016-II | 2.0 (1.0 - 4.0) | 194,291 (124,649 - 260,404) |
| 2016-III | 3.0 (1.0 - 5.5) | 196,522 (115,000 - 266,726) |
| 2016-IV | 2.0 (1.0 - 5.0) | 174,454 (122,271 - 280,664) |
| 2017-I | 2.0 (1.0 - 4.7) | 193,865 (129,818 - 284,856) |
| 2017-II | 2.0 (1.0 - 5.0) | 172,051 (117,471 - 283,283) |
| 2017-III | 2.0 (1.0 - 4.0) | 180,316 (121,054 - 268,730) |
| 2017-IV | 2.0 (1.0 - 4.8) | 177,861 (123,805 - 252,777) |
| 2018-I | 2.0 (1.0 - 4.0) | 162,826 (125,361 - 254,898) |
| 2018-II | 2.0 (1.0 - 4.0) | 176,205 (125,678 - 269,512) |
| 2018-III | 2.0 (1.0 - 4.0) | 165,105 (121,467 - 269,948) |
| 2018-IV | 2.0 (1.0 - 5.0) | 174,807 (126,175 - 275,443) |
| 2019-I | 2.0 (1.0 - 4.0) | 172,700 (122,057 - 269,506) |
| 2019-II | 2.0 (1.0 - 4.0) | 165,664 (122,083 - 251,913) |
| 2019-III | 2.0 (1.0 - 4.0) | 183,022 (130,627 - 264,004) |
| 2019-IV | 1.0 (1.0 - 5.0) | 172,995 (130,436 - 273,621) |

*Median (interquartile range).

**
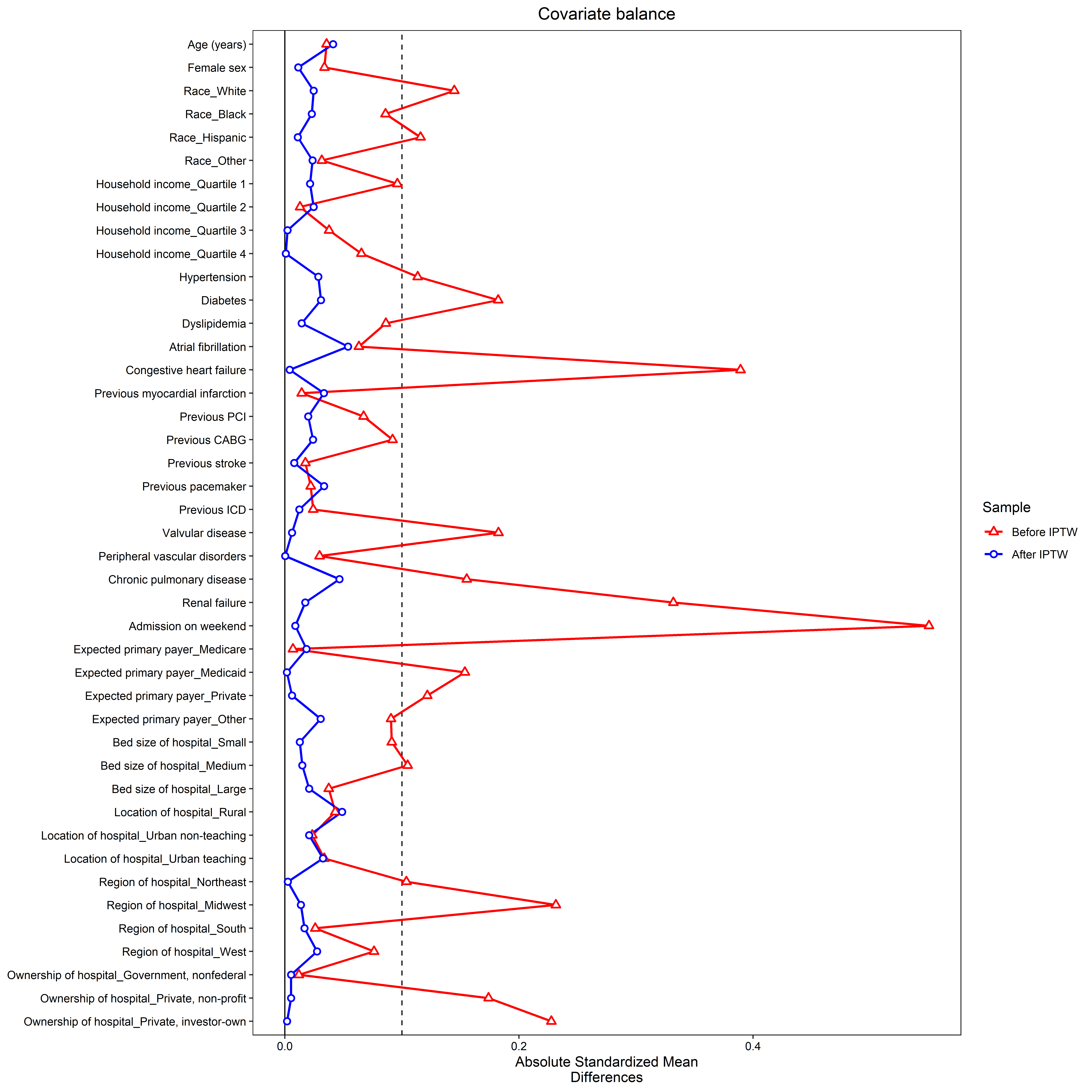
**

**Figure S1. Balance in baseline covariates before (red line) and after (blue line) the inverse probability of treatment weighting.**

**
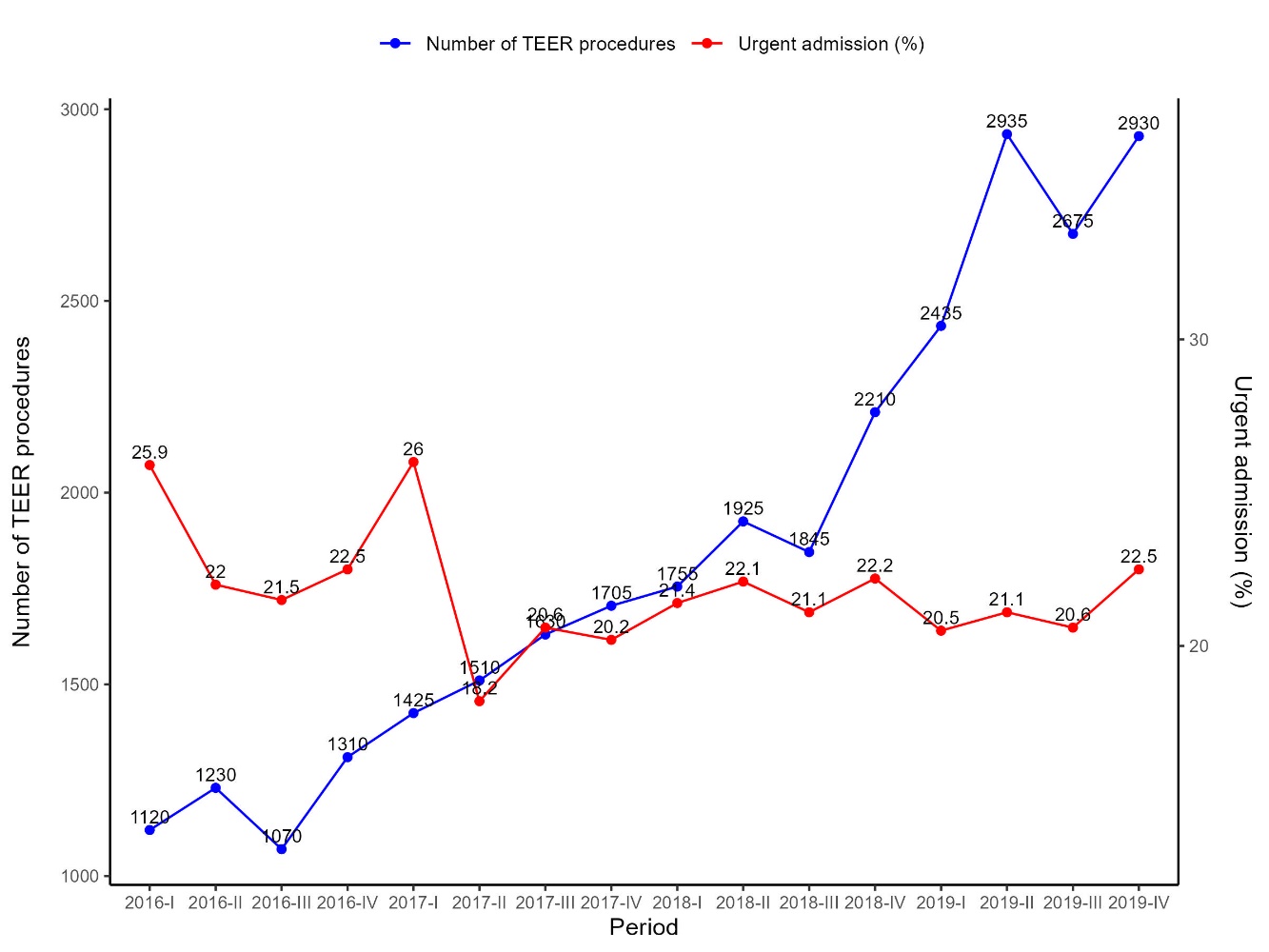
**

**Figure S2. Trend plot of TEER procedures and urgent TEER admissions.**
